# Hydrogel Extrusion Speed Measurements for the Optimization of Bioprinting Parameters

**DOI:** 10.3390/gels10020103

**Published:** 2024-01-27

**Authors:** Stelian Arjoca, Florina Bojin, Monica Neagu, Andreea Păunescu, Adrian Neagu, Virgil Păunescu

**Affiliations:** 1 Department of Functional Sciences, Victor Babes University of Medicine and Pharmacy Timisoara, 300041 Timisoara, Romania; arjoca.stelian@umft.ro (S.A.); florinabojin@umft.ro (F.B.); neagu.monica@umft.ro (M.N.); vpaunescu@umft.ro (V.P.); 2 Center for Modeling Biological Systems and Data Analysis, Victor Babes University of Medicine and Pharmacy Timisoara, 300041 Timisoara, Romania; 3 OncoGen Institute, 300723 Timisoara, Romania; 4 Carol Davila University of Medicine and Pharmacy Bucharest, 050474 Bucharest, Romania; andreea.paunescu@stud.umfcd.ro; 5 Department of Physics and Astronomy, University of Missouri, Columbia, MO 65211, USA

**Keywords:** extrusion-based bioprinting, pneumatic extrusion, hydrogel flow rate, printing speed

## Abstract

Three-dimensional (3D) bioprinting is the use of computer-controlled transfer processes for assembling bioinks (cell clusters or materials loaded with cells) into structures of prescribed 3D organization. The correct bioprinting parameters ensure a fast and accurate bioink deposition without exposing the cells to harsh conditions. This study seeks to optimize pneumatic extrusion-based bioprinting based on hydrogel flow rate and extrusion speed measurements. We measured the rate of the hydrogel flow through a cylindrical nozzle and used non-Newtonian hydrodynamics to fit the results. From the videos of free-hanging hydrogel strands delivered from a stationary print head, we inferred the extrusion speed, defined as the speed of advancement of newly formed strands. Then, we relied on volume conservation to evaluate the extrudate swell ratio. The theoretical analysis enabled us to compute the extrusion speed for pressures not tested experimentally as well as the printing speed needed to deposit hydrogel filaments of a given diameter. Finally, the proposed methodology was tested experimentally by analyzing the morphology of triple-layered square-grid hydrogel constructs printed at various applied pressures while the printing speeds matched the corresponding extrusion speeds. Taken together, the results of this study suggest that preliminary measurements and theoretical analyses can simplify the search for the optimal bioprinting parameters.

## 1. Introduction

Biofabrication is a dynamic research field that aims to create biological products from live cells, biomolecules, and biocompatible materials. Since native biological systems display a tight link between form and function, the success of biofabrication depends on the precise placement of the ingredients [1]. To this end, one option is three-dimensional (3D) bioprinting, which relies on computer-aided transfer processes for dispensing bioinks into live structures of a prescribed 3D organization [2,3,4]. Bioinks can be composed of clusters of cells or biomaterials (e.g., hydrogels) loaded with cells. Additionally, bioinks may also contain cell-laden microcarriers, nanoparticles, and bioactive compounds [5]. A vast diversity of 3D bioprinting techniques and bioinks are being developed, allowing for a shift of focus from achieving the desired 3D structure to promoting biological function [6]. Nevertheless, an old but versatile technique, extrusion-based bioprinting (EBB), remains widely used [7], and the optimization of the corresponding printing parameters is of continued interest [8,9,10,11].

Parameter optimization requires dozens of experiments to cover commonly used printer settings by changing one parameter at a time [10]. Besides the sheer workload, this task is challenging because the quality of the print is hard to quantify. A close match between the digital design and the bioprinted structure is just one requirement; another is to ensure mild physicochemical conditions for the cells found in the dispensed bioink. The parameter optimization index (POI) proposed by Webb and Doyle is a surrogate measure that takes into account both of the above aspects in the context of EBB [10].

Ruberu et al. took advantage of machine learning to reduce the workload of printing parameter optimization [9]. First, they conducted a random set of experiments and scored the outcomes by a sum of quantitative measures of filament formation and layer stacking. The printer settings and the corresponding scores were used to initialize a Bayesian optimization algorithm. Then, the algorithm recommended the printer settings for a new batch of experiments. Their results were fed back into the algorithm and the cycle was repeated until an optimal print was obtained. Depending on bioink composition, the entire process involved between 4 and 47 experiments [9]. Machine learning was also utilized by Tian et al. to optimize the EBB of composite bioinks made of alginate-gelatin hydrogels loaded with live cells [12]. They trained random forest regression, random forest classification, and linear regression models on a dataset extracted from 75 studies aiming to predict bioprinting outcomes, such as extruded filament diameter and cell viability associated with a given set of printing parameters [12].

Paxton et al. proposed an effective workflow for assessing the printability of bioinks developed for EBB [13]. It was composed of preliminary experiments on bioink extrusion, measurements of bioink rheology, and the mathematical modeling of bioink flow. The latter provided a range of printer parameters in which the given bioink could be dispensed through a cylindrical nozzle. Moreover, the model was used to compute the shear stress experienced by the cells next to the nozzle wall and the time spent by the cells within the extrusion nozzle. These quantities are predictors of cell viability. Embryonic stem cells were found to be affected by shear stress values of a few hundred Pa [14], whereas L929 mouse fibroblasts had a post-printing viability of over 90% at shear stress levels of up to 5 kPa [15].

In their investigations of tissue engineering scaffolds built by 3D printing, Chen et al. applied non-Newtonian hydrodynamics to model the flow rate of colloidal gels dispensed by pneumatic extrusion [16,17,18]. Based on the model, they computed the moving speed of the dispensing head (printing speed) needed to deliver scaffold strands with no tensile or compressive stress.

In EBB, the emerging hydrogel strand’s diameter is larger than the inner diameter of the extrusion nozzle [19]. This phenomenon, known as extrudate swell, die swell, or Barus effect, originates from the elastic recovery of the polymer chains from the composition of the hydrogel as soon as they get past the nozzle. Extrudate swell was first discovered in the 1960s, in the context of extrusion processing of molten polymers, and has been the subject of extensive theoretical and experimental investigations [20,21,22]. Because of extrudate swell, knowledge of the hydrogel flow rate is insufficient for computing the extrusion speed defined as the speed of advancement of the dispensed hydrogel strand, also known as extrusion rate, feed rate, or dispensing velocity.

In the context of in situ crosslinking of photopolymerizable inks, the extrusion speed should match the printing speed [23]. On the other hand, in the case of viscoelastic inks, the mismatch between these speeds can be exploited to obtain a variety of outcomes, ranging from coiling patterns to strands thinner than the inner diameter of the nozzle. The extruded pattern depends on two parameters: the printing speed to extrusion speed ratio and the standoff distance expressed in units of the extruded strand diameter [24]. Therefore, extrusion speed measurements are essential for the control of the resolution of EBB. Recent research suggests that experimental tests of bioink printability [13,25], combined with theoretical modeling [26,27], can guide bioink development and narrow down the search for optimal printer settings.

This study extends the set of preliminary tests of bioink extrudability proposed by Paxton et al. with measurements of bioink flow rate and extrusion speed. Assuming volume conservation, from these quantities we estimate the extent of extrudate swell. Then, we use theoretical models of hydrogel flow rate and extrudate swell ratio to fit the experimental data, and, finally, to compute the printing speed needed to achieve a desired print resolution (dispensed strand diameter). The experiments involved in this study were conducted on Cellink Start (Cellink, Göteborg, Sweden), a commercially available hydrogel, chosen due to its excellent printability and good shape stability; it serves as a reference material for bioink development [28].

## 2. Results and Discussion

### 2.1. Measurements and Theoretical Fit of the Flow Rate of Dispensed Hydrogel

**Figure 1:**
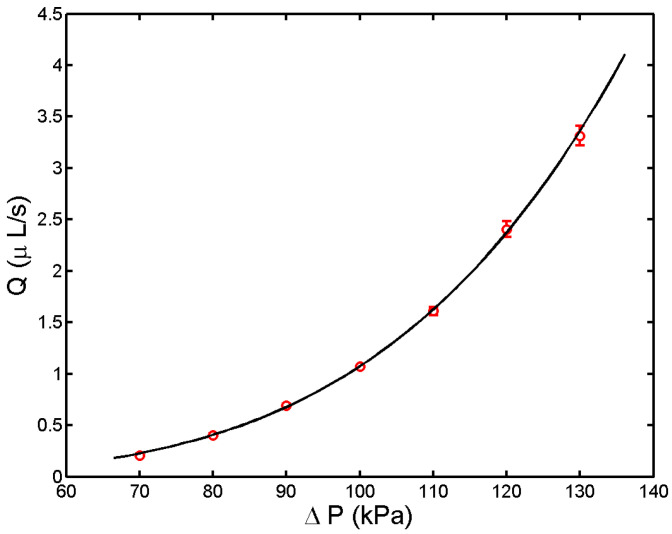
Hydrogel flow rate as a function of the applied pressure. Markers represent the mean values of five independent experiments; error bars show standard deviations, while the solid line plots the result of the best fit based on non-Newtonian hydrodynamics, Equation (1), Section 4.4.

Figure 1 shows the hydrogel flow rate, Q, versus the applied extrusion pressure, ΔP. Circular markers represent the measured values of the volumetric flow rate, whereas the error bars depict the corresponding standard deviations. It can be observed that, with the increase of the extrusion pressure from 70 kPa to 130 kPa, the flow rate increased more than 15-fold. This can be explained by the shear-thinning behavior of the hydrogel.

Several bioinks currently used in EBB display shear-thinning characterized by the Power Law model, $\eta =K{\dot{\gamma }}^{n-1}$, where: η is the bioink’s viscosity (Pa s), $\dot{\gamma }$ is the shear rate (s^−1^), K is the consistency index ($\mathrm{Pa}\;s^{n}$), and n is the power law index (dimensionless, positive, and less than 1) [13]. For Newtonian fluids, Q is proportional to ΔP. In contrast, for shear-thinning fluids that obey the Power Law, Q is a non-linear function of the applied pressure, given by Equation (1) (see Materials and Methods, Section 4.4).

The line of best fit (solid line in Figure 1), was obtained using the *fminsearch* function from MATLAB’s Optimization Toolbox. The fit provided the following material constants for the Cellink Start hydrogel: n=0.23 and $K=222\;\mathrm{Pa}\;s^{n}$.

### 2.2. Extrusion Speed Measurements

**Figure 2:**
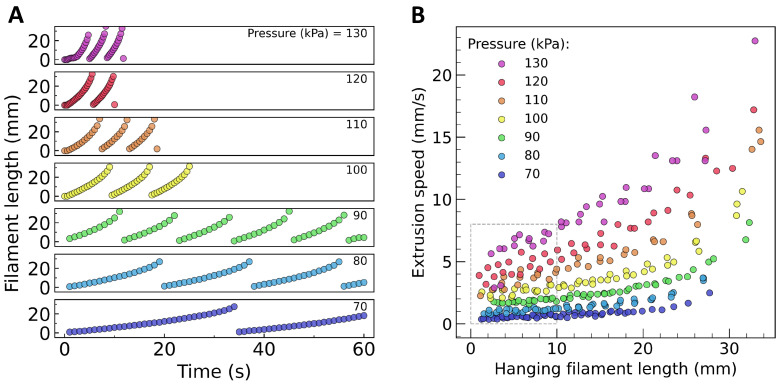
Length and extrusion speed of free-hanging filaments as extracted from video snapshots. (**A**) Extruded filament lengths vs. time recorded at various applied pressures. (**B**) The speed of descent of the extruded filament’s lower extremity plotted vs. filament length. To compute the extrusion speed for a given pressure, we averaged the speeds of advancement recorded before the filament reached 10 mm in length (data points within the dotted rectangle).

By analyzing snapshots from video recordings of hanging hydrogel filament extrusion, we determined the time dependence of the filament length (Figure 2A), as well as the filament tip’s speed of descent at different values of its length (Figure 2B). Overall, it could be observed that as the applied pressure increased, so did the filament extrusion speed, due to the higher flow rate. However, the hydrogel strand’s speed of advancement seemed to be affected by its total length as well. This happens because the strand stretches under its own weight.

Figure 2 indicates that while the filament was short, its own weight did not contribute to its speed of descent. Indeed, the slope of each curve in Figure 2A was roughly constant at the beginning. If the applied pressure suddenly ceased, the hanging hydrogel strand kept its length (not shown). Nevertheless, as the extrusion proceeded, at a certain length, the stress caused by the filament’s weight exceeded the yield stress of the hydrogel. The speed of descent of the extruded filament’s tip increased until the filament eventually ruptured and a new one started to form. In our experiments, ruptures occurred at a strand length of about 30–35 mm, regardless of the applied pressure. Figure 2A shows sequences of filament formation, growth, and rupture, whereas Figure 2B shows the speeds of filament descent at various lengths, pooled together. To calculate the extrusion speed that corresponded to a given applied pressure, we took the arithmetic mean of the speeds of descent of filaments before they reached 10 mm in length.

### 2.3. Theoretical Interpolation of Extrusion Speed Data

**Figure 3:**
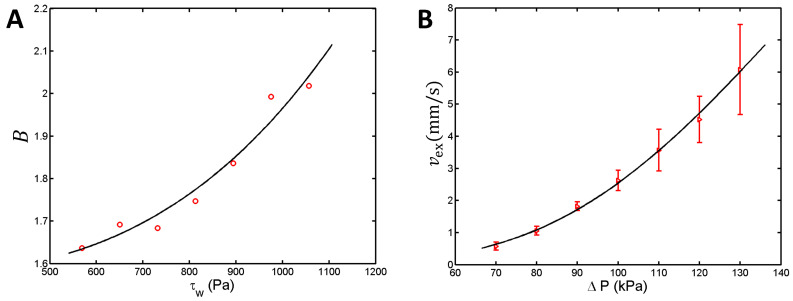
Assessment of the extrudate swell and theoretical interpolation of the extrusion speed values. (**A**) The extrudate swell ratio, $B=R_{\mathrm{ex}}/R$, assessed experimentally (circles) and fitted using Equation (3) from Section 4.4 (solid line). (**B**) Measured and calculated values of the extrusion speed (triangles and solid line, respectively) as a function of the applied pressure, for a 22G extrusion nozzle of 0.5 inch in length. Error bars represent standard deviation.

Experimental measurements of both the flow rate and extrusion speed enabled us to compute the extruded hydrogel strand’s radius, $R_{\mathrm{ex}}$, by assuming that the hydrogel volume is conserved, as per Equation (2) (Section 4.4). The circular markers from Figure 3A plot the extrudate-swell ratio, $B=R_{\mathrm{ex}}/R$, versus the shear stress at the cylindrical nozzle’s wall, $\tau_{w}=R\Delta P/(2L)$. Here, R and L denote the inner radius and length of the cylindrical extrusion nozzle.

It has been established previously that B depends on the composition of the extruded material, nozzle geometry, and extrusion conditions. In the case of polymeric melts, B was found to increase non-linearly with $\tau_{w}$ [29]. To characterize the extrudate swell of the hydrogel, we used Equation (3) from Section 4.4 to fit the extrudate swell data (Figure 3A, solid line), and obtained $c_{1}=$ 1.57, β= 3.15, and $c_{2}=$ 1.38 ×10^−10^ Pa^−*β*^.

The shear stress is maximal near the nozzle wall (see Section 4.4). Since certain cell types can withstand shear stress levels of up to 5 kPa [15], Figure 3A suggests that a bioink of comparable rheological properties could be dispensed through a 22G cylindrical nozzle without significant harm to the embedded cells.

We further combined Equations (1)–(3) to compute the extrusion speed for applied pressures not investigated experimentally. Triangular markers in Figure 3B represent the measured extrusion speed and the whiskers show the corresponding standard deviations, whereas the solid line is the theoretical estimate of the extrusion speed derived from Equations (1)–(3). Note that no additional fit parameter is responsible for the agreement between experiment and theory. The material constants of the hydrogel were determined previously in the fit procedures that were related to the extrusion rate (Figure 1) and the Barus effect (Figure 3A).

**Figure 4:**
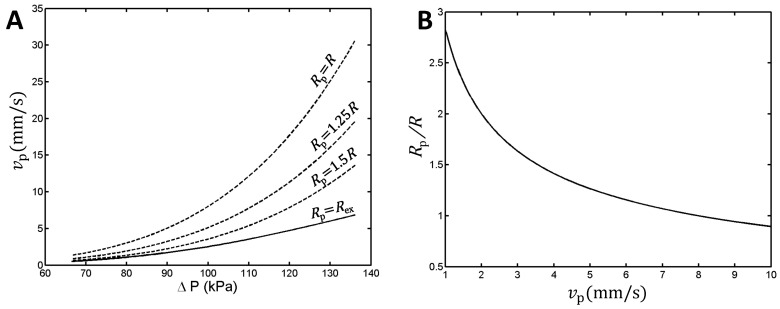
Theoretical estimate of the printing speed required for a target value of the extruded strand diameter. (**A**) The solid line represents the extrusion speed ${(v}_{p}=v_{\mathrm{ex}})$ calculated as a function of the pressure, ΔP, exerted by the pneumatic print head; dashed curves plot the printing speed vs. applied pressure for desired values of the extruded strand radius, $R_{p}$, expressed in terms of the radius of the nozzle’s orifice, R. (**B**) The ratio of extruded strand thickness to nozzle diameter vs. printing speed at an applied pressure of 100 kPa. For calculations, we used Equations (1)–(4) (Section 4.4) applied for a 22G needle (R= 0.2065 mm and L=12.7 mm), and the material constants derived from the fits represented in Figure 1 and Figure 3A.

Finally, Equations (1) and (4) enable one to compute the printing speed needed for a desired hydrogel strand thickness (Figure 4).

The above estimates, however, rely on the assumption that the hydrogel volume is conserved and that the strand does not break down while being deposited. Jungst et al. suggested that tuning the printing speed can compensate for the Barus effect to a certain extent [19]. Indeed, Figure 4 indicates that a full compensation of the Barus effect required printing speeds that were several times higher than the extrusion speed. For example, at an extrusion pressure of 100 kPa, the extrusion speed was 2.6 mm/s, whereas for $R_{p}=R$ one needed a printing speed of 8 mm/s.

Once the material constants are determined for a given hydrogel or bioink, the theoretical model can be used to express the parameter optimization index (POI), defined as the ratio of print accuracy to theoretical shear stress. The POI was proposed as a standardized metric for the assessment of printing parameters in EBB [10]. High values of POI indicate optimal bioprinting conditions, in which the cells experience low shear stress while the bioink is dispensed as a thin strand, ensuring both high cell viability and excellent geometric accuracy. In the modeling framework of our study, $POI=1/\left({2R}_{p}\tau_{w}\right)$.

Taken together, the above results suggest that, besides the hand-extrusion assessment proposed by Paxton et al. [13], bioink developers might use the flow rate and extrusion speed measurements combined with the theoretical modeling to characterize bioink rheology and extrudate swell. Then, the print resolution can be evaluated by increasing the printing speeds beyond the extrusion speed until the dispensed filament breaks down. This workflow can reduce the experimental effort and material costs involved in bioprinting parameter optimization studies [10].

### 2.4. Experimental Validation of Extrusion Speed Assessments

**Figure 5:**
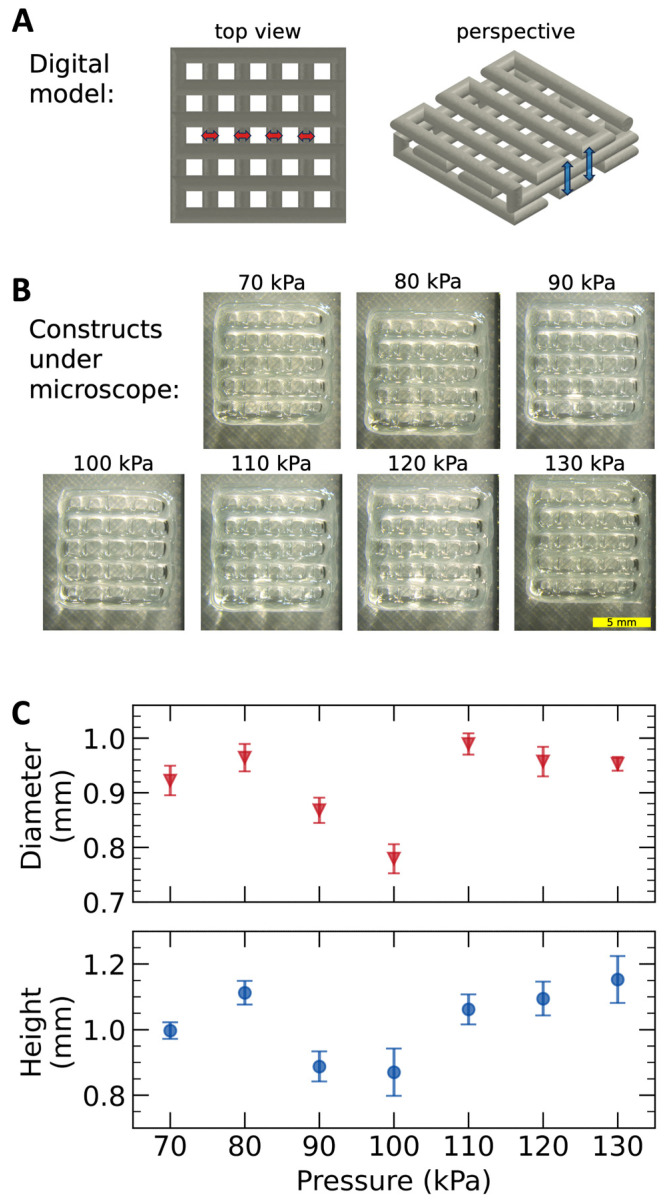
Experimental validation of extrusion speed assessments. (**A**) A digital model of the hydrogel construct (red and blue arrows represent the segments used for the determination of mean filament diameter and mean construct height, respectively). (**B**) Top view photographs of the bioprinted structures visualized using a Leica M205 FA stereomicroscope (scale bar = 5 mm). (**C**) Deposited filament diameter and construct height were obtained at different applied pressures while the printing speed matched the estimated extrusion speed (i.e., the hydrogel filament was not stressed while being deposited).

For the experimental validation of extrusion speed assessments, we started with the digital design shown in Figure 5A. We manufactured this model using different extrusion pressures (ranging from 70 to 130 kPa, in 10 kPa increments) and printing speeds that were set equal to the corresponding extrusion speeds. Thus, we hypothesized that the bioprinter would deposit structures of similar dimensions with slight differences in strut diameters arising from the more pronounced extrudate swell observed at higher shear stress.

Top-view photographs of the fabricated constructs, captured by a stereomicroscope, are presented in Figure 5B. Figure 5C provides a comparison of the structures in terms of filament diameter at specific points and construct height (as marked by red and blue arrows, respectively, in panel A). Based on these metrics, the structures appeared remarkably similar, with less than a 20% variation in filament width and less than a 10% variation in construct height.

Construct height was measured from the side view of the constructs placed on a 3D-printed support featuring a 45° angled mirror to capture both the top view and side view of the hydrogel construct. Supplementary Figures S1 and S2 show stereomicroscopy pictures of the constructs obtained at extrusion pressures of 70 kPa and 130 kPa, respectively.

Despite the extrusion pressure almost doubling during the experiment and the printing speed increasing more than 10 times, the observed variation in filament thickness remained below 20%. This increase in strut diameter, however, was less than expected, given that the extrudate swell ratio, B, increased by 25.2% as the applied pressure increased from 70 kPa to 130 kPa (Figure 3A). Furthermore, the thicknesses of hydrogel strands extruded at pressures of 90 kPa and 100 kPa deviated from the increasing trend observed at other pressures. It is not clear whether these discrepancies can be ascribed to random errors arising from evaporation, gel inhomogeneity, or deposited filament fusion due to surface tension.

The present study has several limitations. First, it only deals with pneumatic extrusion-based bioprinters outfitted with cylindrical nozzles. The flow rate of shear-thinning hydrogels through tapered nozzles has been found numerically by Sarker and Chen [18]. Thus, the methodology outlined in this work may be also applied to conical nozzles, but with a higher computational load. Second, our experiments were conducted on a single hydrogel, Cellink Start, with excellent printability and good shape stability. Cellink Start is not light-curable and dissolves in a cell culture medium; therefore, it is not recommended for building live tissue constructs [30]. This work is a proof-of-concept study, not an actual bioprinting investigation. Further experiments will be needed to demonstrate its practical importance for common bioinks—photopolymerizable hydrogels loaded with live cells at various concentrations. Indeed, several studies indicate that high cell density favors the maturation of the bioprinted tissue construct, but alters the rheological properties of the embedding hydrogel, and, thereby, the printability of the bioink [31].

## 3. Conclusions

This paper proposed a methodology for adjusting the printing speed in pneumatic extrusion-based bioprinting. It requires measuring both the volumetric flow rate and the extrusion speed of free-hanging hydrogel strands dispensed by an immobile print head. The dispensed strand diameter can be computed from the volume conservation. Because of the extrudate swell, it is larger than the inner diameter of the nozzle. An empirical formula can be used to characterize the extent of extrudate swell, and formulas derived from non-Newtonian hydrodynamics can be applied to calculate the extrusion speed for applied pressures not investigated experimentally. Finally, the theoretical analysis provides the printing speed needed for a desired deposited strand diameter (provided that the strand does not rupture as the printing speed exceeds the extrusion speed).

The blend of experimental and theoretical tools developed in this work might help minimize the trial and error approach to establishing optimal bioprinting parameters. Nevertheless, further investigations will be needed to evaluate the proposed approach in the context of extrusion-based bioprinting of bioinks with variable cell density.

## 4. Materials and Methods

### 4.1. Bioprinting Conditions

In this study, we used a BIO X bioprinter (Cellink, Göteborg, Sweden) equipped with a standard pneumatic print head. Due to its low cost and good homogeneity and printability, we chose to use the Cellink Start hydrogel (Cellink, Göteborg, Sweden) during our experiments. Cellink Start is a water-soluble, poly(propylene oxide)-based hydrogel meant to be used as temporary support material for dispensing bioinks of poor shape fidelity [30]. Its rheological properties have been thoroughly characterized in a recent study dedicated to the development of pentanoate-functionalized hyaluronic acid hydrogels [28].

The Cellink Start hydrogel allowed us to perform the experiments at room temperature, without the need for precise temperature control. Nevertheless, using the air conditioning equipment of our laboratory, the room temperature was maintained between 20 °C and 25 °C.

The bioprinter’s print head was equipped with a cylindrical nozzle: a 22-gauge (22G) blunt needle half an inch in length and 0.413 mm in inner diameter (Cellink, Göteborg, Sweden).

### 4.2. Flow Rate Determination

To determine the flow rate corresponding to each applied pressure (from 70 kPa to 130 kPa, in 10 kPa increments), we continuously extruded hydrogel filaments for 60 s. These filaments were collected in glass Petri dishes (Cellink, Göteborg, Sweden), and weighed using a Sartorius TE153S analytical balance (Sartorius AG, Göttingen, Germany; readability of 0.001 g). Five determinations were performed for each pressure and the results were reported in terms of mean value and standard deviation.

### 4.3. Extrusion Speed Measurements

The experiments outlined in Section 4.2. were filmed from a fixed distance using the CCD camera of a smartphone (Apple iPhone 13 Pro, Apple, Inc., Cupertino, CA, USA) mounted on a tripod. The videos were captured at a frame rate of 60 Hz and in high resolution (3840 × 2160 pixels). Examples of all extrusion pressures under consideration are provided in the Supplementary Materials (Video S1).

We processed the recorded videos with the SnapMotion software version 5.0.9 to extract frames at 250 ms intervals. These snapshots were then imported into the ImageJ software version 1.53k [32] to measure the length of the hanging filaments at various time points. Pixel size was established by using the needle’s length as a calibration reference. The filament extrusion speed was determined by assessing the change in filament length between consecutive time points in relation to the corresponding time interval. For each applied pressure, we focused only on extrusion speed evaluations until the occurrence of the second rupture of the hanging filament. As the filaments extended in length and gained weight, their speed of advancement rose because of elongation caused by gravity (as can be observed in Video S1 and Figure 2). For this reason, we limited the analysis only to the data points corresponding to filaments of up to 10 mm in length, where gravitational elongation was minimal. In this interval, the filament extrusion speed was calculated for each applied pressure and expressed as mean value and standard deviation.

### 4.4. Theoretical Estimates of the Extrusion Speed Based on Non-Newtonian Hydrodynamics

Hydrogel rheology was described using the Power Law model to express viscosity, η, in terms of shear rate, $\dot{\gamma }$, as follows: $\eta =K{\dot{\gamma }}^{n-1}$. Here K is the consistency index, and n is the power law index [13]. For the theoretical modeling, we considered a bioprinter with a pneumatic print head that extruded a hydrogel strand through a cylindrical nozzle of length L and inner radius R, under an applied pressure ΔP. According to non-Newtonian hydrodynamics, the hydrogel’s volumetric flow rate is given by (for details, see, e.g., [33], Ch. 6]):

(1) $$Q=\pi {\left(\frac{\Delta P}{2KL}\right)}^{\frac{1}{n}}\frac{R^{3+\frac{1}{n}}}{3+\frac{1}{n}}$$

In this study, we measured the flow rate (as explained in Section 4.2) and used Equation (1) to determine the rheological parameters, K and n, of the Cellink Start hydrogel. We computed these material constants from a non-linear least-squares fit based on the Nelder-Mead simplex search method, as implemented in the *fminsearch* function from the Optimization Toolbox of MATLAB R2014a (The MathWorks, Natick, MA, USA).

Furthermore, we measured the extrusion speed, $v_{\mathrm{ex}}$, (Section 4.3), and applied volume conservation to calculate the average radius, $R_{\mathrm{ex}}$, of the extruded hydrogel filament:

(2) $$R_{\mathrm{ex}}=\sqrt{\frac{Q}{\pi v_{\mathrm{ex}}}}$$

To characterize the extrudate swell of the hydrogel used in our experiments, we used the Curve Fitting Toolbox from MATLAB to fit the experimental data with the empirical formula,

(3) $$R_{\mathrm{ex}}/R=c_{1}+c_{2}\tau_{w}^{\beta }$$

established previously for composite melts of polypropylene filled with diatomite [29]. In the context of our experiments, $\tau_{w}=R\Delta P/(2L)$ ([33], Ch. 6.)

We also interpolated the extrusion speed data by expressing $R_{\mathrm{ex}}$ from Equation (3), and using Equations (1) and (2) to compute $v_{\mathrm{ex}}$ for values of ΔP not tested experimentally.

If the printing speed, $v_{p}$, (defined as the print head’s velocity with respect to the print bed) matches the extrusion speed, the deposited filament’s radius is expected to be near $R_{\mathrm{ex}}$, apart from the deformation caused by gravity and its interactions with the receiving substrate or previously printed filaments. If $v_{p}$ is larger than $v_{\mathrm{ex}}$, the hydrogel strand is stretched while being deposited and the printed strand radius, $R_{p}$, will be smaller than $R_{\mathrm{ex}}$. From volume conservation, we find:

(4) $$R_{p}=\sqrt{\frac{Q}{\pi v_{p}}}=R_{\mathrm{ex}}\sqrt{\frac{v_{\mathrm{ex}}}{v_{p}}}$$

We used Equations (1) and (4) to compute the printing speed needed to achieve a target value of the deposited filament radius, $R_{p}$.

### 4.5. Validation of the Extrusion Speed Assesment

To experimentally validate the extrusion speed estimations described previously, we considered the printing of a square lattice structure (the digital design of this structure is shown in Figure 5A). As for the programming of the bioprinter, we used in-house developed Python scripts that allowed us to automatically generate the G-code instructions to create such constructs. Supplementary Video S2 illustrates the simulated toolpath of the bioprinter for creating the desired structures.

For each applied pressure, we programmed the print head movement feed rate to match the corresponding values of the extrusion speeds determined experimentally. In these conditions, we aimed to compare the size of the fabricated structures.

The constructs were printed using Cellink Start hydrogel on rectangular glass plates. Subsequently, these structures were observed and photographed through the optics of a Leica M205 FA stereomicroscope equipped with a Leica DFC450 C Digital Microscope Camera (Leica Microsystems, Wetzlar, Germany). The constructs were placed on a custom support featuring a 45° angled mirror that allowed us to photograph the printed construct from the top and the side simultaneously (as exemplified in Figures S1 and S2). After importing the microscopy images into ImageJ, we determined the width of the suspended filaments at four specific points (marked with red arrows in Figure 5A), as well as the overall height of the structure at two specific locations (marked with blue arrows in Figure 5A).

## Data Availability

All data and materials are available on request from the corresponding author. The data are not publicly available due to ongoing researches using a part of the data.

## Supplementary Materials

The following supporting information can be downloaded at: https://www.mdpi.com/article/10.3390/gels10020103/s1, Figure S1: Photographs of the triple-layered square-grid hydrogel construct deposited under an applied pressure of 70 kPa; Figure S2: Photographs of the triple-layered square-grid hydrogel construct deposited under an applied pressure of 130 kPa; Video S1: Extrusion of free-hanging hydrogel filaments through cylindrical 22G nozzles at various applied pressures (video playback speed = 5×); Video S2: Simulation of bioprinter tool path to deposit the triple-layered square-grid constructs.
