# Supplementary material for: Hydrogel Extrusion Speed Measurements for the Optimization of Bioprinting Parameters
Source: Gels. 2024 Jan 27;10(2):103. doi: 10.3390/gels10020103 (PMC10888060; doi:10.3390/gels10020103)
Supplement: Supplementary file 1 [file gels-10-00103-s001.zip › Supplementary_figures.pdf]

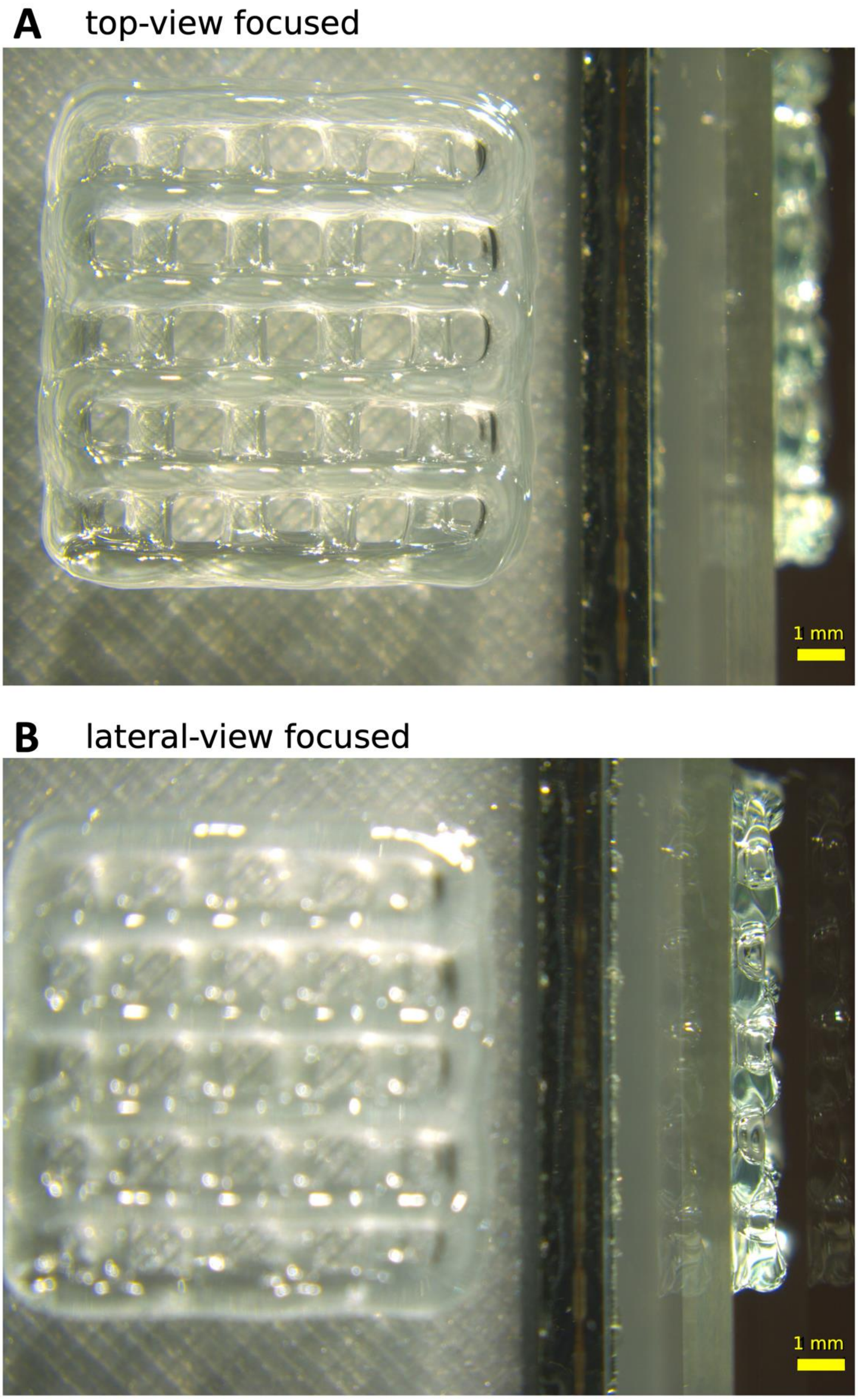

Figure S1. Photographs of the triple-layered square-grid hydrogel construct deposited under an applied pressure of 70 kPa. The stereomicroscopy image is focused on (A) the top view and (B) the lateral view of the structure (scale bar = 1 mm).

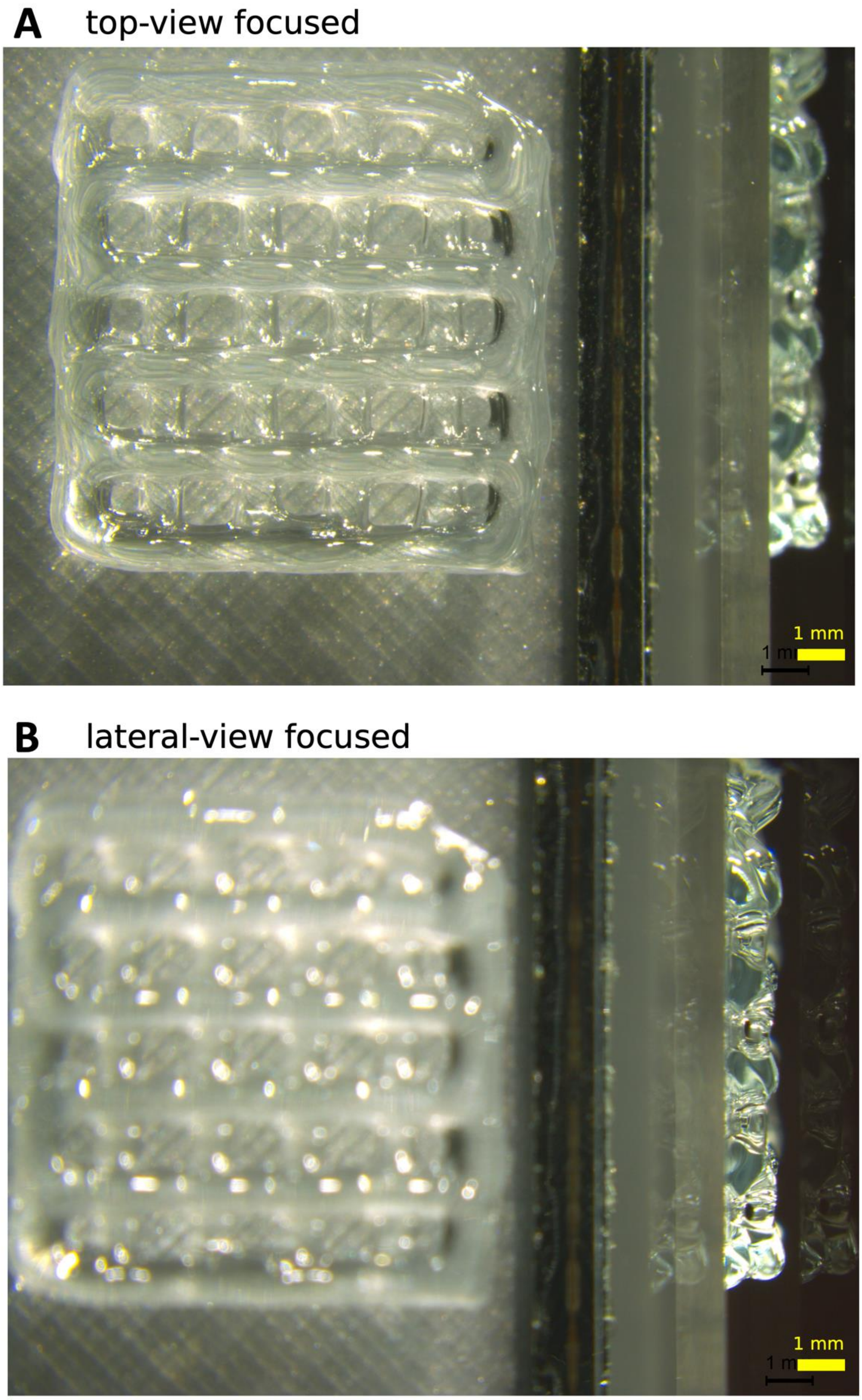

Figure S2. Photographs of the triple-layered square-grid hydrogel construct deposited under an applied pressure of 130 kPa. The stereomicroscopy image is focused on (A) the top view and (B) the lateral view of the structure (scale bar = 1 mm).
